# Metronomic vinorelbine is an excellent and safe treatment for advanced breast cancer: a retrospective, observational study

**DOI:** 10.7150/jca.60682

**Published:** 2021-07-03

**Authors:** Chien-Ting Liu, Meng-Che Hsieh, Yu-Li Su, Chaio-Ming Hung, Sung-Nan Pei, Chun-Kai Liao, Yu-Fen Tsai, Hsiu-Yun Liao, Wei-Ching Liu, Chong-Chi Chiu, Shih-Chung Wu, Shih-Ho Wang, Ching-Ting Wei, Kun-Ming Rau

**Affiliations:** 1 Division of Hematology-Oncology, Department of Internal Medicine, Kaohsiung Chang Gung Memorial Hospital, Kaohsiung 833, Taiwan.; 2 Chang Gung University, College of Medicine, Tao-Yuan 333, Taiwan; 3 Department of Hematology-Oncology, E-Da Cancer Hospital, Kaohsiung 822, Taiwan.; 4 College of Medicine, I-Shou University, Kaohsiung 822, Taiwan.; 5 Department of Surgery, E-Da Cancer Hospital, Kaohsiung 822, Taiwan.; 6 Department of Surgery, Kaohsiung Chang Gung Memorial Hospital, Kaohsiung 833, Taiwan.; 7 Division of General Surgery, Department of Surgery, E-Da Hospital, Kaohsiung, 822 Taiwan.

**Keywords:** metastatic breast cancer, advanced breast cancer, metronomic chemotherapy, vinorelbine, effect.

## Abstract

Advanced breast cancer (ABC) has become a chronic disease. In such a situation, an effective therapy with low toxicities and economically acceptable is needed. Metronomic vinorelbine (mVNR) has been proved to be effective on the control of MBC. The aim of this study is to evaluate the efficacy and safety of mVNR as the salvage therapy for patients with ABC. Oral vinorelbine (VNR) was administered at 70 mg/m2, fractionated on days 1, 3, and 5, for 3 weeks on and 1 week off. Once the mVNR was combined with trastuzumab, or was combined with bevacizumab, the schedule was changed to 2 weeks on and 1 week off. Clinical data of patients with ABC who had received treatment with mVNR and tumor characteristics were collected and analyzed. From Mar. 2013 to Dec, 2020, there were 90 patients with ABC received mVNR. The overall response rate was 53.3% and overall disease control rate (DCR) was 78.9% in this study, including 4 (4.4%) cases reached complete response, 44 (48.9%) cases reached partial response and 23 (25.6%) cases were table disease. The median time to treatment failure (TTF) of the Lumina A patients was 13.3 months, Lumina B patients was 9.1 months, Her-2 enrich patients was 8.9 months, and triple negative breast cancer (TNBC) patients was 5.6 months. Median overall survival time for Lumina A, Lumina B, Her-2 enrich and TNBC were 54.6 months, 53.3 months, 59.5 months and 24.5 months separately. Side effects were minimal and manageable. Metronomic VNR can be an effective treatment for ABC either works as a switch maintenance or salvage therapy. In combination with target therapy or hormonal therapy, mVNR can further improve TTF and DCR with minimal toxicities. Further study should focus on the optimal dosage, schedule and combination regimen.

## Introduction

Advanced breast cancer (ABC), including metastatic breast cancer (MBC) and locally advanced breast cancer (LABC) are generally incurable diseases. However, because of improvements in systemic therapies, the overall survival time of ABC patients is getting longer. As a palliative setting for ABC, good control of tumors, economically acceptable for the healthcare system, and avoidance of toxicity from therapies might be as important as improving survival.

Although recent improvements of hormonal therapies and targeted therapies do provide good tumor control with low toxicity profiles, chemotherapy is still required during the course of treatment in most patients 1. Conventional chemotherapy administers drugs at, or close to, the maximal tolerated dose (MTD). While MTD chemotherapy might kill chemotherapy-sensitive cancer cell populations, the toxicities may deteriorate patients' quality of life (QoL), so a drug-free interval is needed. A break in therapy, however, may allow resistant cells to re-colonize, ultimately leading to disease progression and development of resistance 2.

In contrast to MTD drug regimens, metronomic chemotherapy (MCT) is the continuous administration of drugs at minimally toxic doses without prolonged drug-free intervals. MCT was first introduced in 2000 by Douglas Hanahan, based on preclinical papers from the laboratories of Judah Folkman and Robert Kerbel 3, 4. MCT has an effect on tumor cells, and also the surrounding microenvironment. A fundamental concept of MCT is that tumor cells might acquire resistance to cytotoxic chemotherapy, but acquired resistance would not be expected for vascular endothelial cells 5. In an early experiment, an anti-angiogenic schedule of cyclophosphamide (CTX) increased the apoptosis of endothelial cells that preceded the apoptosis of drug-resistant tumor cells. This metronomic schedule eradicated the majority of drug-resistant Lewis lung carcinomas, and avoided acquired drug resistance when compared with a conventional chemotherapy schedule 6.

Metronomic administration of chemotherapeutic drugs holds a great deal of promise to address several of the major weak points of MTD regimens. These include the development of drug resistance, suppression of anti-tumor immunity, high toxicity, and poor QoL during therapy. The most suitable agents for MCT are oral, inexpensive, well-tolerated, and with no or minimal cumulative toxicity.

Many studies of MCT have been published over the past decades involving patients with ABC. Experimental protocols have often included CTX, methotrexate, capecitabine (CAPE), and vinorelbine (VNR). These drugs have also been associated with hormonal treatments or targeted agents like trastuzumab and bevacizumab 7. Based on these advantages, MCT can be an alternative to standard-schedule chemotherapy or as maintenance therapy in times of remission to delay disease progression 8.

Vinorelbine has been approved for the treatment of breast cancer (BC) and non-small cell lung cancer (NSCLC) in Europe, and for NSCLC only in the United States, alone or in combination with difference chemotherapies 9. VNR is a semi-synthetic vinca-alkaloid that targets tubulin, which prevents tumor cells from making spindles that are needed for division during the cell cycle, and as such has anti-proliferative properties. The recent approval of an oral formulation of VNR has opened the way to development of MCT with this drug. Metronomic VNR (mVNR) had been proven to be well-tolerated, even for elderly patients. Several clinical trials investigating the effect of mVNR on different kinds of cancer have been performed over the last few years 10. A phase IA dose-ranging study confirming that mVNR can safely be administered at doses up to 50 mg three time a week and that it can yield not only long-lasting antitumor effect without overt toxicities, but also lack of drug accumulation 11.

In a study of 34 elderly patients not pretreated for MBC, VNR was administered at 70 mg/m^2^, fractionated on days 1, 3, and 5, for 3 weeks on and 1 week off, every 4 weeks. The overall response rate (ORR) was 38%, median progression free survival (PFS) was 7.7 months, and median overall survival (OS) was 15.9 months 12. For elderly patients with MBC, 30 mg VNR every other day as the first- or further-line treatment, the ORR was 68.7% including 6 complete response (CR, 18.8%) and 16 partial response (PR ,50%). Six patients (18.7%) achieved stable disease (SD) with a disease control rate (DCR) of 87.4%. After a median follow-up of 12 months, median PFS was 9.2 months. The most encouraged finding was few grade 3,4 toxicities were reported in these trials 13.

Because most published data regarding mVNR are from clinical trials, which usually recruit selected patients, the data may not represent real world experience. Thus, in this study we retrospectively collected and analyzed clinical data and treatment outcomes of patients with MBC or LABC who were treated with mVNR, either as a single agent or in combination with other drugs such as hormonal therapy or targeted therapies.

## Methods

### Study Population

Clinical data of patients who received mVNR between March 2013 and Dec. 2020 at 2 hospitals were retrospectively collected and reviewed. The study design was approved by Institutional Review Board of Kaohsiung Chang Gung Memorial Hospital (IRB No. 201700595B0) and E-Da Cancer Hospital (EMRP-110-040).

Inclusion criteria were pathological confirmed ABC, and treated with mVNR. Tumor characteristics such as estrogen receptor (ER), progesterone receptor (PR), and human epidermal growth factor receptor 2 (Her-2) status, Ki-67 expression, and molecular subtype (luminal A, luminal B, Her-2-enriched, and triple-negative breast cancer [TNBC]) were recorded. Metastatic sites including regional lymph nodes and distant organs were also recorded and analyzed.

### Treatment

Vinorelbine was administered at 70 mg/m^2^, fractionated on days 1, 3, and 5, for 3 weeks on and 1 week off, every 4 weeks. This schedule was used if VNR was prescribed as a single agent, or was combined with hormonal therapy for hormonal receptor (HR) positive patients. Patients with Her-2 overexpressed breast cancers also received triweekly trastuzumab. If mVNR was combined with trastuzumab for Her-2+ ABC patients, or was combined with bevacizumab for TNBC patients, the schedule was changed to 2 weeks on and 1 week off, every 3 weeks. Pre-medications included loperamide and prochloperazine, both taken 30 minutes before VNR. Data collected included tumor characteristics, clinical parameters (e.g., site of metastases), treatment events (e.g., number of therapeutic cycles, start/end dates, and rationale for discontinuation), clinical response, use of supportive care medications (e.g., granulocytic colony stimulating factor), dose adjustments, and adverse events. The reported results were based on effectiveness analysis of data collected by Dec. 2020.

### Outcome measures

The primary outcome measure was response rate, including the DCR, which was defined as the proportions of patients who achieved complete response (CR), partial response (PR), and stable disease (SD) as the best response. Tumor response was assessed using Response Evaluation Criteria in Solid Tumors 1.1, with computed tomography scans at baseline. Bone scan and chest X-ray were used as adjuvant evaluation tools. Thus, the percentages of patients with advanced LABC or MBC who achieved CR, PR, and SD during mVNR treatment were recorded. The safety of mVNR was evaluated by the number of patients with adverse events (AEs) and the severity of AEs, which were assessed using the Common Terminology Criteria for Adverse Events (CTCAE), version 4.0. This included all events that were not present before the initial administration of mVNR, pre-existing events that became more intense or more frequent, and events that were present upon initial mVNR administration, but became more severe following administration.

### Statistical analysis

Characteristics of patients and tumors, treatment duration, tumor response, and other categorical variables were summarized as number and percentage, and age as median (range). The time to treatment failure (TTF) was defined as the period from the first dose of mVNR to cancellation for any reason including death, disease worsening, treatment toxicity, patient request, or was censored at the date of last follow-up for surviving patients remaining on treatment. Overall survival time (OS) was defined as the period from the first dose of mVNR to the date of patient death, loss of follow-up or the date as of last follow-up for surviving patients. Univariate analysis was performed to determine the associations of tumor responses (objective response rate [ORR], CR+PR; and DCR, CR+PR+SD) with patient characteristics. The differences of tumor responses were compared using the Pearson chi-square test or Fisher's exact test for categorical variables. Subgroup analysis of TTF was performed using the Kaplan-Meier method and log-rank test assess the associations of TTF and different characteristics. Univariable Cox regression analyses were also used to identify the associations of TTF and different characteristics. All statistical assessments were 2-tailed, and a value of p > 0.05 was considered to indicate statistical significance. All data analysis was performed using Stata Statistical Software (Release 11, StataCorp LP, College Station, TX).

## Results

### Patient characteristics

From Mar. 2013 to Dec, 2020, 90 patients with either LABC or MBC received mVNR, data of most patients were available for toxicity and effectiveness analysis. A summary of patient characteristics is shown in Table 1.

The median age at the time of starting mVNR was 56.0 years old. There were 24 (26.7%) patients what were luminal A, 39 (43.3%) were luminal B, 14 (15.6%) were Her-2 enriched, and 13 (14.4%) patients were TNBC. Forty-three cases (47.8%) were HR+, including 19 cases in luminal B, 34 (36.7%) were Her-2 +, including 20 cases who were HR+ and Her-2 +.

Lung and liver were the most common metastatic organs, and there were 9 (10.0%) patients with only bone metastasis. Most patients had received at least one line of therapy before they began mVNR. The status of switching to mVNR, 45 (50%) patients were PR to the previous regimen, 14(15.6%) patients were SD. For patients whose previous responses were CR, PR or SD, changing to mVNR was like switching maintenance therapy. Twenty-six (28.9%) patients had PD at the time they switched to mVNR.

### Treatment efficacy

The ORR was 53.3% and overall DCR was 78.9% for all patients; 4 (4.4%) patients achieved CR, 44 (48.9%) achieved PR, and 23 (25.6%) had SD (Table 2). Almost all molecular subtypes had a good response except 13 cases of TNBC. Overall response rate for HR+/Her-2 -, Her-2 + (including HR+/Her-2 + and Her-2 enrich) and TNBC were 60.5%, 58.8% and 7.7% respectively. Disease control rate for HR+/Her-2 -, Her-2 + and TNBC were 88.4%, 82.3% and 30.8%. TNBC group was significantly worse than other groups (p<0.001) (Table 2).

**Figure 1:**
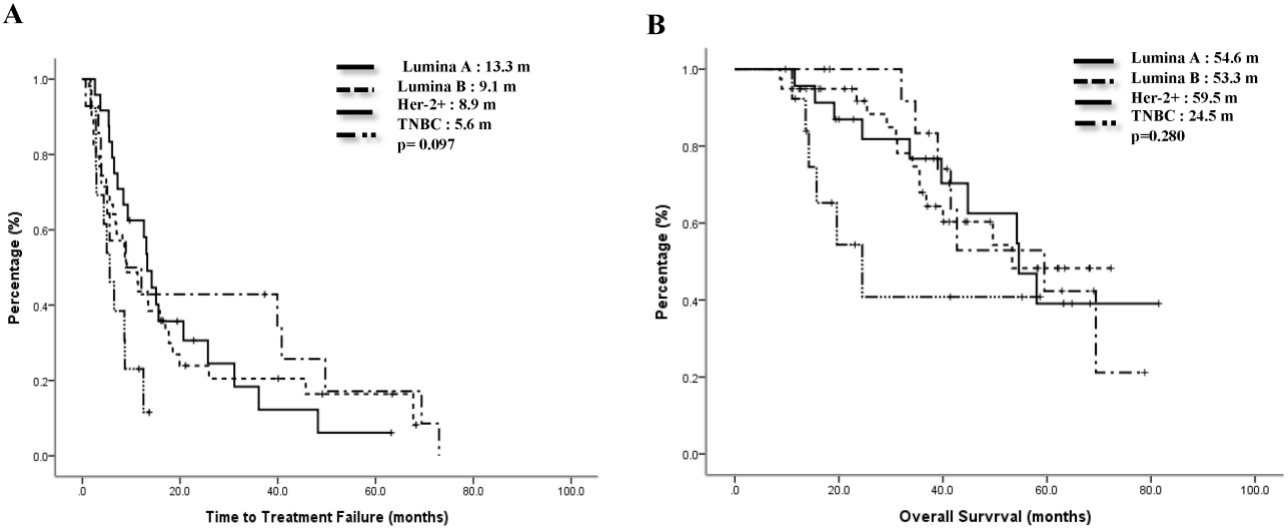
Time to treatment failure (A) and overall survival (B) of all 4 subgroups.

**Figure 2:**
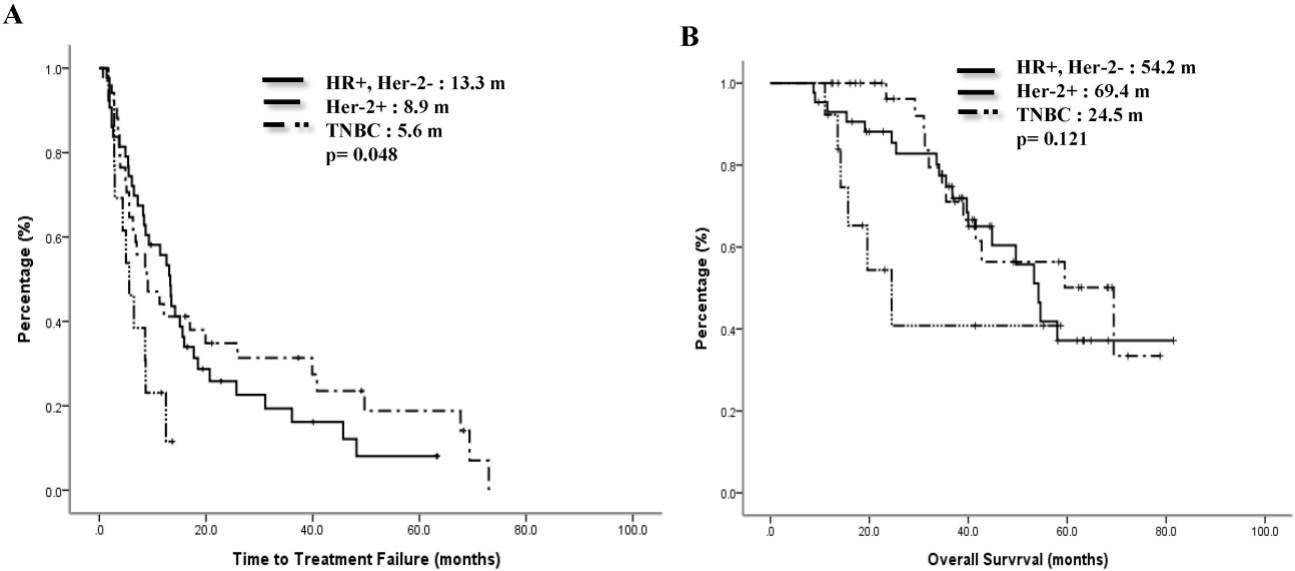
Time to treatment failure (A) and overall survival (B) of 3 groups.

The median TTF of the Lumina A patients was 13.3 months, Lumina B patients was 9.1 months, Her-2 enrich patients was 8.9 months, and TNBC patients was 5.6 months. Compared to the TNBC group, the non-TNBC group had a trend toward better median TTF, p=0.097 (Figure 1A). Median OS for Lumina A, Lumina B, Her-2 enrich and TNBC were 54.6 months, 53.3 months, 59.5 months and 24.5 months separately, p=0.28 (Figure 1B). If we divided patients into 3 groups, the median TTF for HR+/Her-2-, Her-2+ and TNBC were 13.3 months, 8.9 months and 5.6 months, p=0.048 (Fig 2A), median OS were 54.2 months, 69.4 months and 24.5 months separately, p=0.121 (Figure 2B).

**Figure 3:**
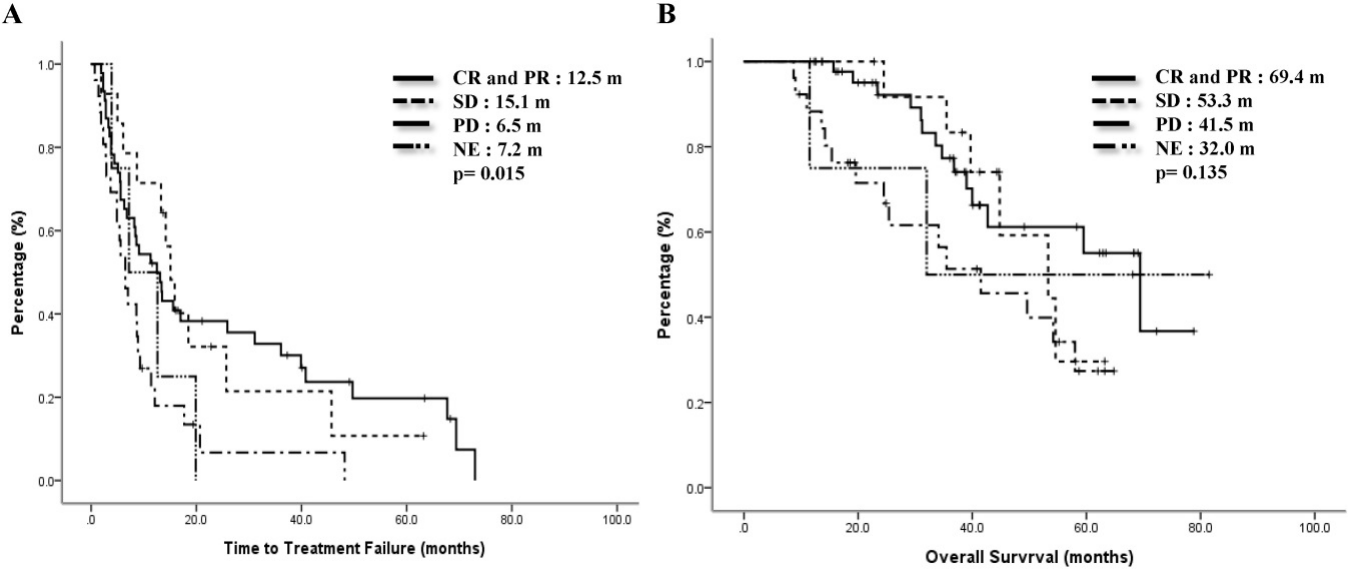
Time to treatment failure (A) and overall survival (B) by response to previous treatment.

Previous treatments might have impacts on the effect of mVNR. For 46 patients whose treatment response to the previous regimen were CR or PR, the ORR to mVNR was 65.5% and DCR was 80.4%; for 14 patients whose previous treatment response was SD, the ORR to mVNR was 42.9% and DCR was 92.9%; and for 26 patients whose previous treatment response was PD, the ORR to mVNR was 46.2% and DCR was 65.4% (p=0.197) (Table 3). Median TTF for previous treatment response was CR/PR, SD, PD and NE were 12.5 months, 15.1 months, 6.5 months and 7.2 months, p=0.015 (Fig 3A) and median OS were 69.4 months, 53.4 months, 41.5 months, and 32.0 months separately, p=0.135 (Figure 3B).

**Figure 4:**
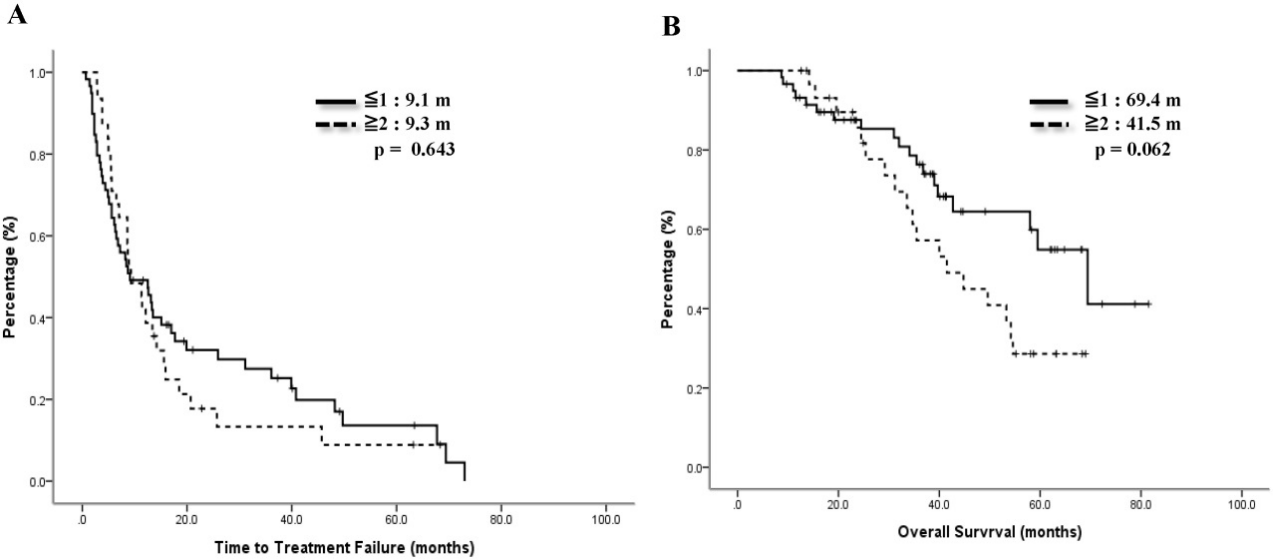
Time to treatment failure (A) and overall survival (B) by treatment linage of vinorelbine.

We also checked the impact of linage of mVNR on therapeutic effects. 59 (65.6%) cases took mVNR as the first or secondary line of therapy, ORR was 61.0% and DCR was 83.0%, including 3 cases of CR. Another 31 (34.4%) cases who tool mVNR after 2^nd^ line of therapy, ORR was 48.3% and DCR was 83.9%. Although more patients got response to mVNR (61.0% vs 48.3%) as the early line, DCR were the same between early and later lines of mVNR (p=.499, Table 4). The median TTF for earlier line was 9.1 months and later line was 9.3 months, p=0.643 (Figure 4A). Median OS for earlier line was 69.4 months versus 41.5 months of the later line, p=0.062 (Figure 4B).

Most patients did not have prominent side effects. The most common side effect was nausea/vomiting, followed by diarrhea and anemia. Leukopenia was not common (Table 5). A large proportion of the patients did not require pre-medications finally. Only 2 patients discontinued mVNR because of side effects. Other patients stopped treatment because of disease progression.

## Discussion

The basic definition of MCT is constant administration of chemotherapy at a low, minimally toxic doses with no prolonged drug-free breaks 14. A meta-analysis of the efficacy and toxicities of MCT for ABC, consisted of 22 clinical trials with 1,360 patients, the pooled ORR was 34.1%, CBR was 55.6%. The overall 6-month PFS, 12-month OS, and 24-month OS rates were 56.8%, 70.3%, and 40.0%, respectively. The pooled incidence of grade 3/4 AEs was 29.5%. Conclusion of this meta-analysis was MCT may be a promising therapeutic method for MBC patients, with a favorable tumor response, survival rate, and low toxicity profile 15.

As an agent of antimicrotubule, vinorelbine can affect adhesion and tight junction of endothelium, intracellular transport of proteins and vesicles, also cell shape and cell polarization. It can also block signaling pathway of vascular endothelium growth factor. Therefore, mVNR can interfere with proliferation and migration of endothelial cells, and inhibit tube formation, sprouting and maintenance of the tumor vasculature and angiogenesis in addition to anti-cancer effect 16. In our practice, we choose oral VNR as the drug for MCT instead of CAP or CTX. The major reason is the flexibility of dosage adjustment and scheduling; patients do not have to take the drug everyday like CTX or CAP 17, 18.

In our study, the ORR of mVNR for all patients was 65.5%, the overall DCR was 80.4%, and the median TTF ranged from 5.6 months for TNBC to 13.3 months for Lumina A. Compared with the results of clinical trials with highly selected patients where the ORR ranged from 17% to 62%, DCR ranged from 24% to 75%, and median PFS was from 3.8 months to 9.82 months, our real world outcome was only inferior to some combination therapy-based MCTs 14. There were no significant differences of median TTF within subtypes, except for TNBC patients. This subtype was the most important risk factor for worse PFS and OS, especially for single agent MCT 19.

In contrast to TNBC, ORR of HR+/Her-2- ABC in our study was 60.5% (Table 2), TTF was 13.3 months and the OS was 54.2 months (Fig. 2). All these patents had combined at least one anti-hormonal agent. Almost in all guidelines for breast cancer treatment, hormonal therapy is not recommended to be combined with chemotherapy because of the possible negative impact on therapeutic effects to each other and no survival benefit 20. Several studies had confirmed that combination of endocrine therapy with MCT were active for MBC 21-23. The targets of metronomic chemotherapy are not only tumor cells but also their microenvironment, especially tumor's neovascularization 6. In SOLTI-1501 VENTANA window of opportunity trial, Adamo et al checked the biological effect of mVNR alone or with letrozole for patients with early breast cancers. They found 3-weeks neoadjuvant mVNR combined with letrozole had superior anti-proliferative effect than both monotherapies. They also found that mVNB differentiated tumor cells into a slightly more estrogen-dependent state, in this context, letrozole was more effective 24.

Currently the choices of secondary line treatment for HR+/Her-2- depend on the previous treatments. In PALOMA3, palbociclib combined with fulvestrant, the ORR was 19%, CBR was 67% 25, updated median PFS was 11.2 months and median OS was 34.9 months 26. In MONARCH-2, abemaciclib combined with fulvestrant, the ORR was 48.1% 27, updated median PFS was 16.9months, and the median OS was 46.7 months 28. In MONALEESA-3, ribociclib combined with fulvestrant, median PFS was 14.6 months and median OS was 40.2 months 29. Comparing to CDK4/6 inhibitors combine with fulvestrant as the secondary line therapy, therapeutic effects of mVNR combined with one anti-hormonal therapy are not inferior to published data, mVNR also has fewer side effects, especially leukopenia, and the price is less expensive than CDK4/6 inhibitor/fulvestrant combination. This issue is more important for patients at developing countries.

Most of time, in the metronomic studies, they chose HR positive tumors, indolent disease, and bone metastases only diseases, Her-2 + ABCs are excluded. In fact, VNR combined with trastuzumab has been shown to be at least as effective as docetaxel and trastuzumab as the first-line therapy of Her-2+ ABC, and has significantly fewer adverse effects 30. In the CLEOPATRA study, patients in each group received docetaxel for a median of 8 cycles 31. The median OS was 57.1 months (95% CI 50-72) in the dual blockade group and 40.8 months (36-48) in the placebo group (hazard ratio 0.69, 95% CI 0.58-0.82) 32. Although trastuzumab/pertuzumab and a taxane-based combination regimen is now the backbone for Her-2 + MBCs, most patients still have to stop taxane because of cumulative toxicities 31. Once chemotherapy is stopped and only anti-Her-2 antibodies are administered, development of resistance is common, especially for HR-negative ABCs. It is reasonable to use mVNR in combination with trastuzumab as a switch maintenance therapy under this situation. Compared with conventional weekly administration, mVNR can achieve the same efficacy but with much fewer side effects. Recently, a phase II study focused on older and frail population with Her-2+ patients of ABC reported that dual blockade of Her-2 plus metronomic chemotherapy provided a better PFS than Her-2 dual blockade alone, and has an acceptable safety profile 33. Fadi et al. reported a phase II study in which oral VNR was given weekly in combination with trastuzumab as the first-line therapy of Her-2+ ABC 34. In their study, the DCR was 88% and the median PFS was 6.7 month, but grade 3/4 hematological toxicities including neutropenia (46%), anemia (4%), and nausea/vomiting (11.5%) were observed.

Our data showed that as a switch maintenance, the ORR of mVNR in Her-2+ group reached 58.8%, DCR was 82.3 %, the median TTF was 8.9 months and the median OS was 69.4months, indicating mVNR can further extend the effect from taxane-based combination (Table 2, Figure 2). Interestingly, all our patients had received taxane before, but none exhibited cumulative neurotoxicity. Because dual blockades are not reimbursed at many countries, it is not easy for patients to receive dual blockade as a maintenance therapy, combination of mVNR with trastuzumab after taxane can be an effective and reasonable choice.

Most of our patients had received at least one line of systemic therapy before they received mVNR. Patients whose diseases were under control by the last treatment (CR and PR) had better ORR, DCR, TTF and OS than those were with PD (65.5% vs 46.2%; 80.4% vs 65.4%; 12.5 months vs 6.5 months and 68.4 months versus 41.5 months; Table 3, Fig. 3). Because most patients had stable or responsive disease when they switched to mVNR, the change was similar to a change in switching maintenance therapy. This switch did provide an alternatively effective therapy, which maintained the previous treatment response, extended the PFS and might be OS in the future, and the toxicities were minimal. The concept of switching maintenance therapy has already been commonly used in patients with lung cancer; when the cancer is under control, a change from a more toxic treatment to a less toxic treatment is made 35.

Whether the effect of mVNR is as effective as standard protocols is under evaluation. The NAME trial, which is a randomized, open-label, parallel, multi-center study, aims to evaluate the efficacy and safety of mVNR versus intermittent oral VNR in patients with Her-2- ABC, and the IBCSG 54-16 trial, a randomized phase II trial of mVNR plus CYC and CAP (VEX) versus weekly paclitaxel as the first-line or second-line treatment in patients with ER +/Her2 - advanced or MBC are ongoing, and the results are awaited in 3-4 years.

In our data, TNBC patients had the worst outcome, the efficacy was similar to other study 36.The reason might be because of the mixed subtypes of TNBC 37. How to improve therapeutic effects for TNBC patients become an important issue. Combination two or more drugs might help to reach better efficiency but no addictive toxicity. The best combination might be metronomic CAP and VNR with a clinical benefit rate near 50% (VICTOR- 1,2). A phase II study assessed the safety and efficacy of metronomic oral chemotherapy with VNR, CYC, and CAP in untreated metastatic TNBC patients. Twenty-two of 25 patients were evaluable for both efficacy and toxicities. The ORR was 27%, CBR was 50%, median TTP was 6.4 months and median OS was 18.4months. Grade ≥3 adverse events were uncommon 36. VICTOR-3 was designed to investigate the role of mVNR, either as a single agent or in combination with metronomic CAP, in TNBC patients after an induction standard-dose CHT, as maintenance therapy is still ongoing 38.

The consensus of a workshop of Italian experts suggested that single agent mVNR can be a treatment choice for HR+/Her-2 - patients with bone or soft tissue involvement, or with visceral metastases but no symptoms, or progressing after a first- or second- line endocrine therapy. The combination of mVNR with target therapy or other chemotherapy such as CAP or CYC appear to be a promising strategy, in order to maintain the benefits deriving from an all-oral regimen, and to avoid hospitalization^39^.

VICTOR-6 study is the largest study reporting data of MCT from real world. Overall response rate of MCT ranged from 33.8% in first-line to 8.8% in forth-line setting. Disease control rate was from 81.5% to 54.4%. Amount all regimens, VNR-based regimens had the highest ORR and DCR in first-line. Overall, median PFS was 7.2 months (95%CI: 5.3-10.3) for VNR-single agent and 9.5 months for VNR-combination; median OS was 22.7 months (95% CI 13.0-43.5) in VNR-single agent and 30.0 (95% CI 26.2-34.7, HR 0.67) for VNR-combination regimens. But in this study, they excluded Her-2 + ABC cases in which subgroup mVNR showed an excellent result as a maintenance therapy in our study 40.

In our study, even mVNR was used at later lines, we still could see almost equally TTF between earlier or later line of therapies for advanced BC, and the data is non-inferior to other drugs like eribulin and pegylated liposomal doxorubicin those are also have been approved after taxane and anthracycline^41^, ^42^. This might be because mVNR targets not only tumor cells, but also endothelial cells, which are believed to not develop resistance to chemotherapy.

Current guideline suggests MCT is a treatment option for patients not requiring rapid tumor response. Available regimens are low-dose oral CYC and methotrexate, capecitabine CAP or oral VNR-based regimens 20. From our study and published data, MCT was associated with fewer toxicities, especially no drug-cumulative side effect which allows for long-term therapy and no need of frequent blood test. Compared with capecitabine which would still lead to skin toxicities, mVNR is lack of drug accumulation over time.

To our knowledge, this retrospective study reported the largest number of patients of Her-2 + ABC patients who receiving mVNR in the real world setting. We found mVNR can be an effective treatment for MBC or LABC of different molecular subtypes. It is also a successful maintenance therapy after intravenous chemotherapy. In combination with targeted therapy, mVNR might improve the therapeutic effectiveness. The side effects of mVNR are minimal and manageable.

## Conclusions

MCT can target endothelial cells to inhibit angiogenesis, and directly kill cancer stem cells and cancer cells, and the effects can be strengthened when used in combination with targeted therapies 43. From our study, metronomic VNR can be an effective treatment for ABC, as either a switch maintenance therapy or a salvage therapy. Due to its good safety profiles, in combination with targeted therapy, other oral chemotherapy, hormonal therapy or even immunotherapy, mVNR can further improve PFS and the DCR without increasing toxicities. Side effects from mVNR are minimal and manageable. Further study should focus on the optimal dosage, schedule, and combination regimen.

## Figures and Tables

**Table 1:** Patients baseline characteristics.

	Number (%)
Median Age at diagnosis, years old (y/o)	50.0
Median age at the time of starting vinorelbine (y/o)	56.0
Molecular subtype	
Lumina A	24 (26.7)
Lumina B	
HR+, Her-2 -	19 (21.1)
HR+, Her-2 +	20 (22.2)
Her-2 enrich	14 (15.6)
Her-2 positive	34 (37.7)
TNBC	13 (14.4)
Initial stage at diagnosis	
I	11 (12.2)
II	25 (27.8)
III	16 (17.7)
IV	38 (42.2)
Metastatic site at vinorelbine	
Lung	45 (50.0)
Liver	28 (31.1)
Brain	8 (7.8)
Bone only	9(10.0)
Skin/Soft tissue	11(12.2)
Others	12 (13.3)
Number of metastatic sites	
1	42 (46.7)
2	22 (24.4)
≥3	26 (28.9)
Previous treatment for MBC	
0	10 (11.1)
1	49 (54.4)
2	17(18.9)
≥3	14(15.6)
Response to previous treatment	
CR	1 (1.1)
PR	45 (50.0)
SD	14 (15.6)
PD	26 (28.9)
NE	4 (4.4)

HR-hormonal receptor; TNBC- triple negative breast cancer; MBC-metastatic breast cancer; CR-complete response; PR-partial response; SD-stable disease; PD-progressive disease; NE-not evaluated.

**Table 2:** Best treatment response in different populations.

Tumor response	CR, n (%)	PR, n (%)	SD, n (%)	DCR, n (%)	PD, n (%)	NE, n(%)
Overall, N=90	4(4.4)	44(48.9)	23(25.6)	71 (78.9)	17(18.9)	2 (2.2)
Subtypes						
Lumina A, N=24	2 (8.3)	15 (62.5)	6 (25.0)	23 (95.8)	1(4.2)	0
Lumina B, HR+, Her-2-, N=19	0	9(47.4)	6(31.6)	15 (78.9)	3 (15.8)	1(5.2)
Lumina B, HR+, Her-2+, N=20	0	12 (60.0)	5(25.0)	17 (85.0)	3 (15.0)	0
Her-2 enrich ,N=14	2 (14.3)	8 (57.1)	3(21.4)	13 (92.9)	1 (7.1)	0
HR +, Her-2 -, N=43	2 (4.7)	24 (55.8)	12 (27.9)	38 (88.4)	4 (9.3)	1 (2.3)
Her-2+, N=34	2 (5.9)	18 (52.9)	8 (23.5)	28 (82.3)	4 (11.8)	1(2.9)
TNBC, N=13	0	1 (7.7)	3 (23.1)	4 (30.8)	9 (69.2)	0

CR-complete response; PD-progressive disease; PR-partial response; SD-stable disease; DCR-disease control rate; NE-not evaluable, HR-hormonal receptor; TNBC-triple negative breast cancer.

**Table 3:** Comparisons of previous treatment response and the best treatment response to mVNR (N=90).

Response to previous treatment	CR, n (%)	PR, n (%)	SD, n (%)	DCR, n (%)	PD, n (%)	NE, n (%)
CR and PR, n=46	1(3.4)	26 (62.1)	10 (24.1)	37 (80.4)	8(3.4)	1(6.9)
SD, n=14	0	6 (42.9)	7 (50.0)	13 (92.9)	1(7.1)	0
PD, n=26	0	12 (46.2)	5 (19.2)	17 (65.4)	8 (30.8)	1 (3.8)
NE, n=4	1(25)	2(50)	1(25)	4 (100)	0	0

CR-complete response; PD-progressive disease; PR-partial response; SD-stable disease; DCR-disease control rate; NE-not evaluable.

**Table 4:** Comparisons of line of treatment and best treatment response with mVNR (N=90).

Linage of previous treatments	CR, n (%)	PR, n (%)	SD, n (%)	DCR, n (%)	PD, n (%)	NE, n (%)
≤1, N=59	3(4.9)	30 (56.1)	12 (22.0)	45 (83.0)	12(12.2)	2 (4.9)
≥2, N=31	1(3.2)	14(45.1)	11 (35.5)	26 (83.9)	5 (16.1)	0

CR-complete response; PD-progressive disease; PR-partial response; SD-stable disease; DCR-disease control rate; NE-not evaluable.

**Table 5:** Side effects from mVNR n (%).

	Gr I	Gr II	Gr III	Gr IV
Nausea/Vomiting	20 (22.2)	16 (17.8)	0	0
Diarrhea	14 (15.6)	7(7.8)	1(1.1)	0
Neutropenia	5(5.6)	5(5.6)	0	0
Anemia	11 (12.2)	5 (5.56)	1 (1.1)	0
Paresthesia	1 (1.1)	2 (2.2)	0	0

mVNR-metronomic vinorelbine; Gr-grade.
